# Fringe Phase-Shifting Field Based Fuzzy Quotient Space-Oriented Partial Differential Equations Filtering Method for Gaussian Noise-Induced Phase Error

**DOI:** 10.3390/s19235202

**Published:** 2019-11-27

**Authors:** Changzhi Yu, Fang Ji, Junpeng Xue, Yajun Wang

**Affiliations:** 1 Institute of Mechanical Manufacturing Technology, China Academy of Engineering Physics, Mianyang 621999, China; yuczcaep@163.com; 2 School of Aeronautics and Astronautics, Sichuan University, Chengdu 610065, China; 3 State Key Laboratory of Information Engineering in Surveying, Mapping and Remote Sensing, Wuhan University, Wuhan 430079, China; yjwangisu@whu.edu.cn

**Keywords:** structured light sensor, image denoising, fringe phase-shifting field, fuzzy quotient space, oriented partial differential equations, phase error

## Abstract

Traditional filtering methods only focused on improving the peak signal-to-noise ratio of the single fringe pattern, which ignore the filtering effect on phase extraction. Fringe phase-shifting field based fuzzy quotient space-oriented partial differential equations filtering method is proposed to reduce the phase error caused by Gaussian noise while filtering. First, the phase error distribution that is caused by Gaussian noise is analyzed. Furthermore, by introducing the fringe phase-shifting field and the theory of fuzzy quotient space, the modified filtering direction can be adaptively obtained, which transforms the traditional single image filtering into multi-image filtering. Finally, the improved fourth-order oriented partial differential equations with fidelity item filtering method is established. Experiments demonstrated that the proposed method achieves a higher signal-to-noise ratio and lower phase error caused by noise, while also retaining more edge details.

## 1. Introduction

Due to the advantages of non-contact, high speed, high precision, three-dimensional (3D) shape measurement, fringe projection profilometry (FPP) [1,2,3,4] has been widely used in industrial detection, quality inspection, etc. Generally, the principle [1,2,3,4,5,6] is to project the sinusoidal and straight fringes onto the surface of the object measured, and then the camera captures the fringe images modulated by the object surface. The shape information of the object can be obtained from the absolute phase according to the phase-height mapping. It is very important to calculate the absolute phase from the fringe images that are captured by the camera. However, fringe signals will be degraded by sensor noise [7,8,9,10] obeying Gaussian distribution, which affects the accuracy of the final three-dimensional measurement information, due to the influence of environmental noise, in the process of image acquisition and transmission. 

As we all know the fringe image contains important information, such as the deformation and displacement of the object measured. Wang [11] analyzed the relationship between phase-shift errors and the accuracy of phase reconstruction, and proposed an accurate phase-shift estimation method. In the past few decades, many studies have been performed on understanding the effect of Gaussian noise on phase reconstruction, which can be roughly divided into two categories, which are, the binary pattern method and filtering method. The basic principle of the binary pattern method [12,13,14,15] is to project binary fringe pattern to improve the peak signal-to-noise ratio (PSNR) of captured fringe images. Wang [15] designed N patterns in an N-dimensional coding space to define and maximize the SNR of pattern. Afterwards, binary pattern method based binary defocusing fringe [13,14] and Gray-code [5,15] were also applied to improve the PSNR of the fringe pattern. However, the edges of binary patterns are often difficult to precisely distinguish, which reduces the measurement accuracy. The filtering method [16,17,18,19,20,21,22,23,24] is to reduce noise by means of filtering algorithms. Image filtering is a process of restoring noise-free image from noise image, in which the difficulty is how to protect details while reducing noise. Therefore, how to filter the Gaussian noise effectively is very important in accurately extracting the phase information. Traditional filtering methods [16,17,18,19,20], such as Gaussian filtering, median filtering, and wavelet transform, were proposed to reduce the noise. Villa [21] proposed a fringe pattern denoising method based on Gaussian convolution to improve the performance of low-frequency fringes. However, unlike general images, the digital fringe image has obvious directional characteristics and it presents sinusoidal distribution. If the traditional filtering methods were used, the edge information of fringe pattern will be blurred while filtering [9], which reduces the accuracy of phase extraction. To this end, partial differential equations (PDEs) methods, such as second-order PDEs, total variation (TV), fourth-order PDEs, oriented PDEs (OPDEs), etc., have been applied for filtering electronic speckle pattern interferometry (ESPI) fringe patterns, which makes the filtering only be carried out along the fringe direction. Tang [20,22,23] proposed a second-order single oriented PDEs method to solve the denoising problems for optical interferometry fringes, and then provided various methods that were based on PDEs. Yang [24] put forward an adaptive model combining the TV and fractional-order differentiation filter for such problems. The second-order PDEs [20,23,24,25] easily lead to the phenomenon of the staircase effect, while the four-order PDEs [26] can effectively reduce the staircase phenomenon and get more attention and application. Recently, through introducing the controlling speed function, Tang [9] established an adaptive oriented PDEs filtering method for discontinuous optical fringe patterns. At the same time, lots of hybrid methods based on PDEs had also been studied, such as fuzzy C-means [27,28], Hessian matrix [29], and shearlet transform [30]. Li [31] proposed a method for multi-frame fringe patterns processing based on convolutional neural network (CNN) in order to extract the fringe skeletons in ESPI. Partial differential equations, especially OPDEs and fourth-order OPDEs, have been demonstrated to be powerful in preserving the details of ESPI fringe patterns while filtering. However, fourth-order OPDEs filtering methods have been widely studied for ESPI fringes, while being rarely applied for digital projection fringe denoising problem. Additionally, we can see that the above methods belong to single image filtering, pursuing the improvement of PSNR of single image. In phase-shifting profilometry (PSP), the phase is determined together by N phase-shifting images. In the presence of random Gaussian noise, the fringe filtering directions at the same location of N phase-shifting images may be different with each other. Traditional single image filtering will often lead to phase shifting.

When considering the limitation above, fringe phase-shifting field based fuzzy quotient space-oriented partial differential equations filtering method (FOPDEs) is proposed for fringe image denoising problem, which provides the fringe image with higher PSNR and reduces the phase error that was caused by Gaussian noise. Firstly, the influence of Gaussian noise on phase error is analyzed. It is concluded that the phase error that is caused by Gaussian noise also presents Gaussian distribution, which can be taken as the theoretical basis of fringe image filtering. Secondly, the concept of fringe phase-shifting field is established to transform the single fringe image filtering into multi-fringe images filtering in the phase-shifting field. Thirdly, the theory of fuzzy quotient space is applied to modify the filtering direction. The pixels along the fringe direction are adaptively clustered, and the filtering direction is corrected according to the phase-shifting field by judging the intensity of the pixel noise. Finally, the improved OPDEs filtering method with fidelity term is proposed, which preserves more details while filtering Gaussian noise. The experimental results show the effectiveness of the proposed FOPDEs method when compared with other methods.

Section 2 introduces the phase error that is caused by Gaussian noise. Section 3 explains the principle of the proposed method. Section 4 shows the experimental performance of the proposed method, and Section 5 summarizes the paper.

## 2. Gaussian Noise-Induced Phase Error

This section will briefly review the FPP and explain how Gaussian noise effects the phase. The projected sinusoidal fringe pattern is presented as [1,2,3,4],

(1) $$\begin{array}{l} I\left(x,y\right)=I_{A}\left(x,y\right)+I_{B}\left(x,y\right)\cos\left[\varphi \left(x,y\right)+\delta_{n}\right]\\ \delta_{n}=\frac{k\;*\;2\pi }{N},N=3,4,5\cdots ,n=0,1,\cdots ,N-1 \end{array}$$

where (x,y) is any point, $I_{A}$ is the average intensity, $I_{B}$ is the intensity modulation, φ is the phase to be solved for, and *N* is the number of phase-shifting steps. According to the *N*-step phase-shifting algorithm, the phase value solved can be described as,

(2) $$\varphi \left(x,y\right)=-\arctan\left[\frac{\sum_{n=1}^{N}I_{n}\left(x,y\right)\sin\delta_{n}}{\sum_{n=1}^{N}I_{n}\left(x,y\right)\cos\delta_{n}}\right]$$

The phase value ranges (−π,π] with 2π discontinuities due to the use of arctangent function. Phase unwrapping algorithms [2,3,4] need to be applied for the continuous phase, which can be used to analyze and compare the phase accuracy of different filtering algorithms in the presence of noise. Assume that the noise-free image *f’* is a real function that is defined on a bounded and piecewise smooth open subset (image domain) $\Omega \in {\mathbb{R}}^{2}$, that is, f:Ω→ℝ. Moving to a discrete formulation of the problem, we assume that $f,f^{\prime }\in {\mathbb{R}}^{k}$ are the function values at the *k* nodes of an equidistant two-dimensional grid of size M×N on Ω. Denote by *f* the noisy image, which can be obtained from image *f’* and the addition of Gaussian noise,

(3) $$f=f^{\prime }+g$$

where *g* represents the additive Gaussian white noise with zero mean and standard deviation *σ*. Thus, given the noisy image *f*, we are interested in recovering *f’*, which is well known to be an ill-posed problem, in general.

In this paper, the noise model that was developed by [31,32] is adopted to quantitatively analyze the noise-induced phase error in phase-shifting algorithm. Usually, the noise is far less than the projected intensity. Therefore, the effect of noise on the phase reconstruction can be regarded as a little perturbation on the measured phase, which leads to the following first-order approximation of the variance of phase error,

(4) $$\sigma_{\varphi }^{2}=\sum_{n=0}^{N-1}\left[\left(\frac{\partial \varphi }{\partial I_{n}}\right)\sigma^{2}\right]$$

Let *ω* be the total periods number in the fringe pattern, substituting Equation (1) into Equation (4), and the variance of phase error can be written as,

(5) $$\sigma_{\varphi }^{2}=\sum_{n=0}^{N-1}\left[{\left(-\frac{2}{N\omega^{2}I_{B}}\sin\left(\varphi -\frac{2n\pi }{N}\right)\right)}^{2}\right]=\frac{2\sigma^{2}}{N\omega^{2}I_{B}^{2}}$$

According to Equation (5), the variance of phase error primarily depends on four factors, the number of phase-shifting steps *N*, the Gaussian noise variance *σ*, intensity modulation *I_B_*, and the fringe density *ω*. Thus, through increasing the number of phase-shifting steps, denser fringe patterns, and higher intensity modulation, noise in phase reconstruction can be reduced. However, for a given high speed or real-time measurement system, where the measured object, measurement time, and number of fringe pattern are fixed, the noise-induced phase error is difficult to suppress or eliminate completely. For a given measurement system, Equation (5) suggests that the Gaussian noise-induced phase error still obeys for Gaussian distribution. The phase error will be included in the absolute phase φ, which will cause the error of object reconstruction according to the mapping of phase-height [2]. We can see that the phase error that is caused by Gaussian noise influences the accuracy of the reconstruction results.

**Figure 1:**
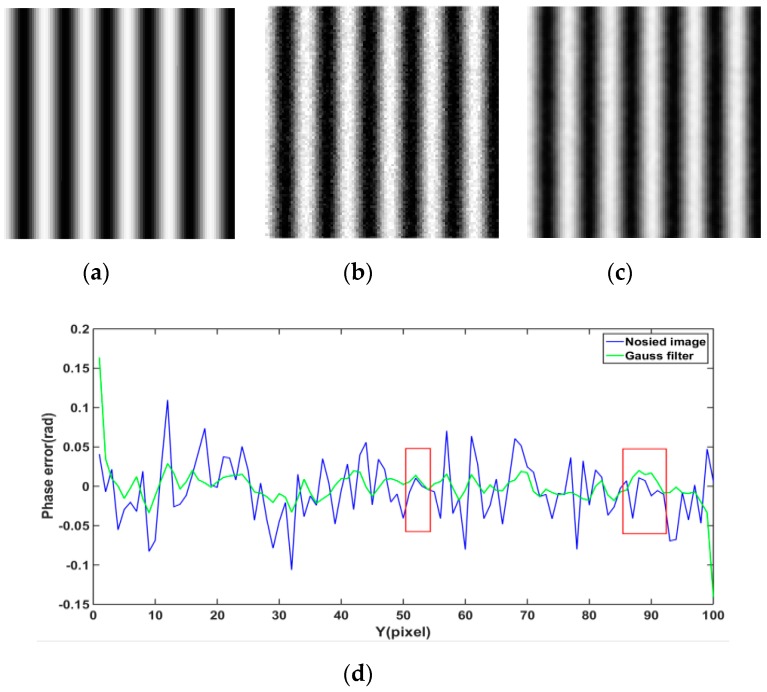
Fringe patterns. (**a**) Noise-free fringe pattern, (**b**) fringe pattern with $\sigma^{2}=0.3\%$, (**c**) fringe pattern with Gaussian filtering, and (**d**) the 25th row cross sections of phase error of noised image and fringe image filtered by Gaussian filtering.

Figure 1a is the ideal fringe pattern, Figure 1b is the fringe pattern with Gaussian noise variance 0.3%, and Figure 1c is the Gaussian filtering result of Figure 1b. The 25th row cross sections of phase error that are shown in Figure 1d are the results of the by Gaussian filtering of Figure 1b,c, minus the ideal phase of Figure 1a. Traditional filtering methods only focus on improving the PSNR of single image, and neglect the effect of filtering on phase reconstruction. From Equation (2) and the red rectangular box of Figure 1d, it can be seen that the phase error that is caused by noise cannot be reduced by only increasing the PSNR of single image. In this work, the main purpose of image filtering is to reduce the phase error that is caused by noise.

## 3. Principles

This section introduces the proposed filtering method to get high PSNR fringe image for phase extraction in order to reduce the influence of Gaussian noise on phase reconstruction.

### 3.1. Fringe Phase-Shifting Field 

The fringe gradient reflects the intensity change of the pixels. Generally speaking, for ideal noise-free vertical fringes, the fringe gradient is along the sinusoidal direction, that is, the horizontal direction. However, being affected by random noise, the gradient direction of the fringes might change, not following the horizontal direction, but showing an angle with the horizontal direction. The gradient vector at point (x,y) can be written as,

(6) $$\nabla f={\left[G_{x},G_{y}\right]}^{Τ}={\left[\frac{\partial f}{\partial x},\frac{\partial f}{\partial y}\right]}^{Τ}$$

where, $G_{x}=\frac{\partial f}{\partial x},G_{y}=\frac{\partial f}{\partial y}$ are the gray change rates of f(x,y) along *x* direction and *y* direction, respectively, which can be expressed by the central difference method. ∇f is a vector, which points to the direction of the maximum rate of change of f(x,y), and its modulus can be expressed as,

(7) $$\left|\nabla f\right|={\left[G_{x}^{2}+G_{y}^{2}\right]}^{\frac{1}{2}}={\left[{\left(\frac{\partial f}{\partial x}\right)}^{2}+{\left(\frac{\partial f}{\partial y}\right)}^{2}\right]}^{\frac{1}{2}}$$

The difference between the fringe patterns and common image is that the fringe images have directional characteristics and they present sinusoidal characteristics along the vertical direction of fringe. We define the perpendicular direction to the fringe gradient direction as the fringe direction, which obeys the right-hand theorem. Let θ be the angle between the fringe direction and the *x*-axis,

(8) $$\theta =\arcsin\frac{\left|G_{x}\right|}{\sqrt{G_{x}^{2}+G_{y}^{2}}}$$

**Figure 2:**
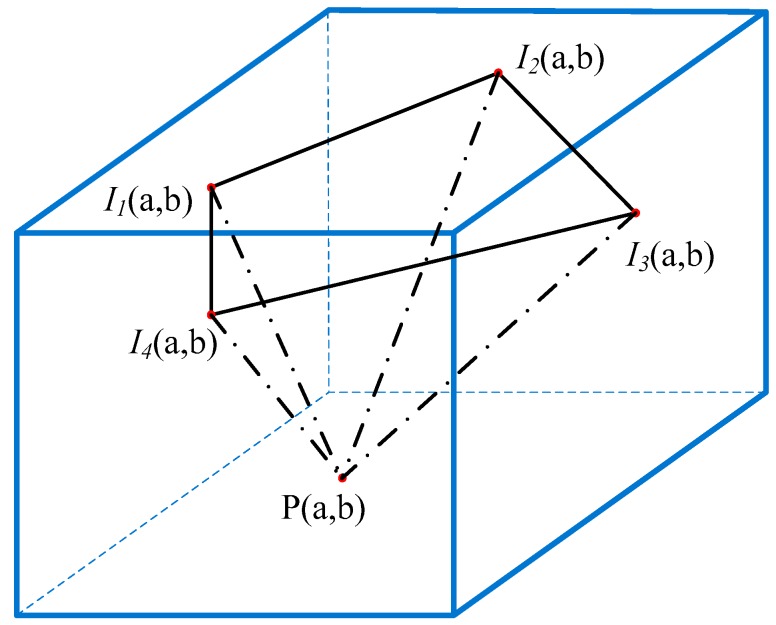
Fringe phase-shifting field (*N* = 4).

The space that consists of *N*-step phase-shifting images is defined as the fringe phase-shifting field, as shown in Figure 2. *I*_1_, *I*_2_, *I*_3_, and *I*_4_ are the projection intensities of the corresponding point (*a*, *b*) in four-step phase-shifting images, respectively. *p* is the phase value of point (*a*, *b*) in the phase map. Next, we give two hypotheses and prove them.

**Hypothesis** **1.** *Assuming that the projected fringe patterns are all standard sinusoidal fringes, and after modulating on the smooth and diffuse reflective surface of the object, for any point of phase map, the fringe direction of the corresponding point is identical with each other in the four-step fringe phase-shifting field*.

**Proof.**  For the four-step phase-shifting method, the intensity at any point (*a*, *b*) can be expressed as $I\left(a,b\right)=I_{A}+I_{B}\cos\left(\phi +\frac{2n\pi }{4}\right),n=1,2,3,4$. $\theta_{1},\theta_{2},\theta_{3},\theta_{4}$ represent the fringe direction angles of the corresponding point of four phase-shifting images, respectively. With the central difference method, the direction angles can be expressed as,

$$\begin{array}{ll} \theta_{1} & =\arctan\frac{I_{1}\left(a+1,b\right)-I_{1}\left(a-1,b\right)}{I_{1}\left(a,b+1\right)-I_{1}\left(a,b-1\right)}\\ & =\arctan\frac{\cos\left(\phi_{a+1,b}+0\right)-\cos\left(\phi_{a-1,b}+0\right)}{\cos\left(\phi_{a,b+1}+0\right)-\cos\left(\phi_{a,b-1}+0\right)}\\ & =\arctan\frac{\cos\left(\phi_{a+1,b}\right)-\cos\left(\phi_{a-1,b}\right)}{\cos\left(\phi_{a,b+1}\right)-\cos\left(\phi_{a,b-1}\right)} \end{array},\;\begin{array}{ll} \theta_{2} & =\arctan\frac{I_{2}\left(a+1,b\right)-I_{2}\left(a-1,b\right)}{I_{2}\left(a,b+1\right)-I_{2}\left(a,b-1\right)}\\ & =\arctan\frac{\cos\left(\phi_{a+1,b}+\frac{\pi }{2}\right)-\cos\left(\phi_{a-1,b}+\frac{\pi }{2}\right)}{\cos\left(\phi_{a,b+1}+\frac{\pi }{2}\right)-\cos\left(\phi_{a,b-1}+\frac{\pi }{2}\right)}\\ & =\arctan\frac{\cos\left(\phi_{a+1,b}\right)-\cos\left(\phi_{a-1,b}\right)}{\cos\left(\phi_{a,b+1}\right)-\cos\left(\phi_{a,b-1}\right)} \end{array},$$

$$\begin{array}{ll} \theta_{3} & =\arctan\frac{I_{3}\left(a+1,b\right)-I_{3}\left(a-1,b\right)}{I_{3}\left(a,b+1\right)-I_{3}\left(a,b-1\right)}\\ & =\arctan\frac{\cos\left(\phi_{a+1,b}+\pi \right)-\cos\left(\phi_{a-1,b}+\pi \right)}{\cos\left(\phi_{a,b+1}+\pi \right)-\cos\left(\phi_{a,b-1}+\pi \right)}\\ & =\arctan\frac{\cos\left(\phi_{a+1,b}\right)-\cos\left(\phi_{a-1,b}\right)}{\cos\left(\phi_{a,b+1}\right)-\cos\left(\phi_{a,b-1}\right)} \end{array},\;\begin{array}{ll} \theta_{4} & =\arctan\frac{I_{4}\left(a+1,b\right)-I_{4}\left(a-1,b\right)}{I_{4}\left(a,b+1\right)-I_{4}\left(a,b-1\right)}\\ & =\arctan\frac{\cos\left(\phi_{a+1,b}+\frac{3\pi }{2}\right)-\cos\left(\phi_{a-1,b}+\frac{3\pi }{2}\right)}{\cos\left(\phi_{a,b+1}+\frac{3\pi }{2}\right)-\cos\left(\phi_{a,b-1}+\frac{3\pi }{2}\right)}\\ & =\arctan\frac{\cos\left(\phi_{a+1,b}\right)-\cos\left(\phi_{a-1,b}\right)}{\cos\left(\phi_{a,b+1}\right)-\cos\left(\phi_{a,b-1}\right)} \end{array}.$$

As can be seen from the above, $\theta_{1}=\theta_{2}=\theta_{3}=\theta_{4}$. That is, for four-step phase-shifting images, the fringe direction of corresponding point is identical with each other. □

**Hypothesis** **2.** *In the four-step fringe phase-shifting field, if the fringe patterns are degraded by Gaussian noise, for one point of phase map, the fringe direction of the corresponding point might be different from each other.*

**Proof.**  According to the Hypothesis 1, for four-step phase-shifting images, the fringe direction of any corresponding point is identical under ideal conditions. However, due to the existence of noise, the random noise intensity at any point is different. In the presence of additive Gaussian white noise $I^{n^{\prime }}$, we assume that the intensity of light at any point (a,b) is expressed as $I\left(a,b\right)=I_{A}+I_{B}\cos\left(\phi +\frac{2n\pi }{4}\right)+I^{n^{\prime }},n=1,2,3,4$; thus, the fringe direction can be rewritten as,

(9) $$\begin{array}{ll} \theta^{\prime } & =\arctan\frac{I_{1}\left(a+1,b\right)-I_{1}\left(a-1,b\right)}{I_{1}\left(a,b+1\right)-I_{1}\left(a,b-1\right)}\\ & =\arctan\frac{I_{B}\cos\left(\phi_{a+1,b}+0\right)+I_{\left(a+1,b\right)}^{n^{\prime }}-I_{B}\cos\left(\phi_{a-1,b}+0\right)-I_{\left(a-1,b\right)}^{n^{\prime }}}{I_{B}\cos\left(\phi_{a,b+1}+0\right)+I_{\left(a,b+1\right)}^{n}-I_{B}\cos\left(\phi_{a,b-1}+0\right)-I_{\left(a,b-1\right)}^{n}}\\ & =\arctan\frac{\cos\left(\phi_{a+1,b}\right)-\cos\left(\phi_{a-1,b}\right)+\left(I_{\left(a+1,b\right)}^{n^{\prime }}-I_{\left(a-1,b\right)}^{n^{\prime }}\right)/I_{B}}{\cos\left(\phi_{a,b+1}\right)-\cos\left(\phi_{a,b-1}\right)+\left(I_{\left(a,b+1\right)}^{n^{\prime }}-I_{\left(a,b-1\right)}^{n^{\prime }}\right)/I_{B}} \end{array}$$

It can be seen from Equation (9) that the random Gaussian noise leads to the deviation of fringe direction. Therefore, for the same corresponding point, fringe directions might be different from each other. According to Hypothesis 1 and 2, the fringe direction could be deviated due to the influence of random Gaussian noise. If the traditional method is applied for filtering, the phase shifting will still occur. Therefore, it is necessary to modify the fringe direction to obtain a more accurate filtering direction. □

### 3.2. Filtering Direction Correction Method Based on Fuzzy Quotient Space

In this subsection, the proposed filtering direction correction method is introduced in detail. The basic idea is that only the strong noise points along the fringe direction are filtered to avoid blurring the weak noise points, so as to better protect the fringe edges. This subsection briefly explains the theory of fuzzy quotient space [33,34,35,36], which classifies the pixels along the vertical direction of the fringe gradient into weak noise point clusters and strong noise point clusters. Some basic notions and properties of fuzzy quotient space theory are given, as follows.

**Definition** **3.** *Let S represents the set of all fuzzy subsets on fringe image domain*Ω*. Assume that function*R∈S(Ω×Ω)*, and satisfies (i) reflexivity,*∀x∈Ω,R(x,x)=1;*(ii) symmetry,*∀x,y∈Ω,R(x,y)=R(y,x);*(iii)*$\forall x,y,z\in \Omega ,R\left(x,z\right)\geq \sup_{y}\left(R\left(x,y\right),R\left(y,z\right)\right).$*R is called a fuzzy equivalence relation on*Ω.

**Proposition** **4.** *Assume that R is a fuzzy equivalence relation on*Ω*and*$R_{\lambda }=\left\{\left(x,y\right)|R\left(x,y\right)\geq \lambda ,\lambda \in \left[0,1\right]\right\}$*. Let*Ω(λ)*be a quotient space corresponding to equivalence relation*$R_{\lambda }$*. It can be seen that*$0\leq \lambda_{2}\leq \lambda_{1}\leq 1\Leftrightarrow R_{\lambda_{1}}>R_{\lambda_{2}}\Leftrightarrow \Omega \left(\lambda_{2}\right)$*is a quotient space of*$\Omega \left(\lambda_{1}\right)$*. A family*{Ω(λ)|λ∈[0,1]}*of quotient spaces forms an order-chain based on the inclusion relation of quotient sets, called hierarchical structure on*Ω.

**Theorem** **5.** *Given a fuzzy equivalence relation R on*Ω*, we have a corresponding hierarchical structure on*Ω.

**Theorem** **6.** *Assume that*{Ω(λ)|λ∈[0,1]}*is a hierarchical structure on*Ω*, there is a fuzzy equivalence relation R on*Ω.

**Proposition** **7.** *Let R be a fuzzy equivalent relation on* Ω *. All the pixels along the fringe direction can be represented by fuzzy equivalent granules* $\left(G_{1},G_{2},\cdots ,G_{i},\cdots ,G_{m}\right)$ *, where* $G_{i}$ *represents a fuzzy sub-granular.* $\forall G_{i},G_{j},d\left(G_{i},G_{j}\right)=1-s\left(a,b\right),\forall a\in G_{i},b\in G_{j}$ *,* d *is a distance function,* $s\left(x_{i},x_{j}\right)$ *indicates the similarity function.*

**Figure 3:**
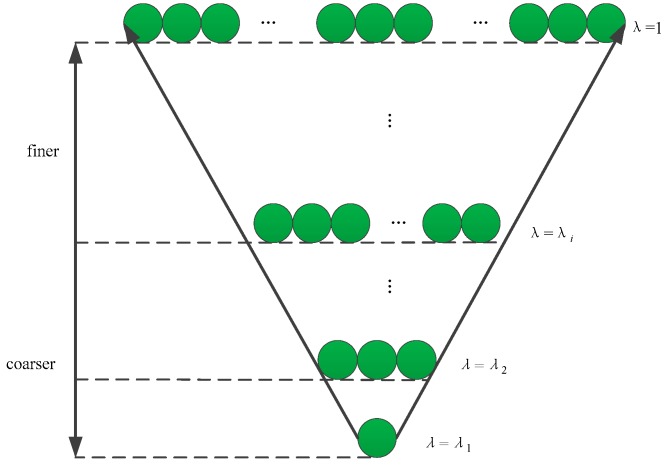
A hierarchical structure.

According to Proposition 7, when given a distance, we can have a fuzzy equivalence relation and a hierarchical structure on Ω, as shown in Figure 3. We define an Euclidean distance of pixels as $d=\mathrm{abs}\left(A\left(x_{i},y_{i}\right)-A\left(x_{j},y_{j}\right)\right)/255$. Thus, a fuzzy similarity matrix *r* with reflexivity and symmetry is constructed as,

(10) $$r_{N\times N}=\left[\begin{array}{ccccccc} 1 & & & & & & \\ \vdots & \ddots & & & & & \\ s_{i1} & \cdots & 1 & & & & \\ \vdots & & \vdots & \ddots & & & \\ s_{j1} & \cdots & s_{ji} & \cdots & 1 & & \\ \vdots & & \vdots & & \vdots & \ddots & \\ s_{N1} & \cdots & s_{Ni} & \cdots & s_{Nj} & \cdots & 1 \end{array}\right]$$

Based on the theory of fuzzy quotient space, triple ${\left(\Omega ,A,G\right)}_{\lambda }$ is used to describe the granulation problem. Ω denotes the whole pixels along fringe direction. *A* denotes the gray value attribute of pixel. *G* denotes the set of attribute values. All of the pixels along the fringe direction can be divided into several fuzzy equivalent granules according to *A*. Subsequently, the union of all granules can represent the domain Ω, that is, the pixels along fringe direction. According to Proposition 7, Ω is a hierarchical structure about granularity λ. In Figure 3, each green circle represents a granule. For different granularities, all of the pixels can be divided into different fuzzy equivalent clusters. That is to say, different clustering results can be obtained by changing the granularity. The granularity is the coarsest when all of the pixels are regarded as one granule. The granularity gets finer with the increase of the number of granules. Therefore, through the fuzzy quotient space, pixels clustering problems can be expressed as the different results at different granularity layers.

As shown in Figure 3, a variety of classification results at different granularity levels can be obtained by the hierarchical structure [35,36,37]. How to extract the optimal granularity layer from the hierarchical structure will be discussed below. Effective granularity layer can better reflect the clustering effect of granules, that is, the inner distance of granule is as small as possible, while the outer distance between the granules is as large as possible. Therefore, the sum of inter- granule distances is defined as inter-granule compactness, and the sum of outer-granule is defined as outer-granule separation. The optimum granularity layer has the highest degree of inter-granule compactness and outer-granule separation. The criteria for judging the optimal granularity layer are as follows,

(11) $$opt(\lambda )=\max\left(\frac{Sim_{avg}}{Dis_{avg}}\right)$$

where $Sim_{avg}$ is the average inter-granule similarity under granularity λ and $Dis_{avg}$ is the average outer-granule distance under granularity λ. Assume that there has *m* granules under granularity λ and $C_{i}^{\lambda }$ represents the *i*th granule,

(12) $$Sim_{avg}=\frac{1}{m}\sum_{i=1}^{m}\frac{\sum_{\left|C_{i}^{\lambda }\right|}s\left(X,Y\right)}{\left|C_{i}^{\lambda }\right|},X,Y\in C_{i}^{\lambda },X\neq Y$$

A granule is a set of many pixels and ${d^{\prime }}_{i}^{\lambda }$ is the average gray value of the granule. The distance between granules can be calculated according to the Euclidean distance, as mentioned above. The average outer-granule distance can be written as,

(13) $$Dis_{avg}=\frac{1}{m}\sum_{i,j=1}^{m}d^{\prime }(i,j),\;i,j\in m,i\neq j$$

Hence, the optimal granularity layer is selected out from the hierarchical structure. According to Section 3.1, noise will cause the deviation of fringe direction. If the filtering process is along the deviated fringe direction, it will still lead to phase shifting, which will ultimately affect the accuracy of phase extraction.

**Definition** **8.** If there is a weak noised point in the four-step phase-shifting field, and its 3*3 neighborhood points are all weak noised points, then this point is called a valid point.

The fringe direction of any corresponding point is $\theta =\left(\theta_{1},\theta_{2},\theta_{3},\theta_{4}\right)$, the filtering direction determination method is as follows,

(1) If there is only one valid point, the fringe direction of the valid point is taken as the filtering direction, that is, $\theta =\theta_{i}$.
(2) If there are two or three valid points, the mean fringe direction of the valid points is taken as the filtering direction, that is, $\theta =Mean\left(\theta_{i},\theta_{i}^{\prime }\right)$ or $\theta =Mean\left(\theta_{i},\theta_{i}^{\prime },\theta_{i}^{\prime \prime }\right)$.
(3) If there is no valid point, the mean value of the fringe direction is taken as the filtering direction, that is, $\theta =Mean\left(\theta_{1},\theta_{2},\theta_{3},\theta_{4}\right)$.

### 3.3. Improved Fourth-Order Opdes Filtering Method

In this subsection, we present an improved fourth-order OPDEs filtering method with fidelity item. The basic idea of image filtering based on partial differential equations theory is as follows. Let $I:R^{2}\to R$ represent a gray image. Additionally, I(x,y) is the gray value of pixel (x,y). Introducing time parameter *t*, then the image evolution process can be expressed as [8,20,21],

(14) $$\partial_{t}u=F\left[u\left(x,y,t\right)\right],u\left(x,y,0\right)=I\left(x,y\right)$$

where u(x,y,t) is the evolutionary image. F:R→R is an operator given for different image processing processes. The original image I(x,y) can be regarded as the initial condition. Thus, the solution of differential equations u(x,y,t) is the image that is processed under the time parameter *t*. The principle of PDEs denoising is transforming the image denoising problem into a minimal functional problem according to the variational framework. Let *E* and $E_{s}$ represent the energy function and the smoothing item, respectively. To preserve more details while filtering, based on the fourth-order OPDEs energy function model of literatures [8,20,21] and Equation (14), we introduce the fidelity item $E_{f}$ to obtain an improved fourth-order OPDEs filtering model, which can be defined as,

(15) $$E\left(u\right)=E_{s}\left(u\right)+E_{f}\left(u\right)=\int_{\Omega }\frac{1}{2}{\left|\frac{\partial^{2}u}{\partial \rho^{2}}\right|}^{2}dxdy+\frac{\lambda }{2}{\left\|u_{0}-u\right\|}_{L^{2}}^{2}$$

where ρ represents the fringe direction of the image u(x,y), that is, the diffusion direction. λ is a constant, which reflects the fidelity of the original image and the denoised image. $u_{0}$ is the original image, while u is the denoised image under time parameter *t*. $L^{2}$ represents the Euclidean norm. The relationship between the coordinate position (x,y) and ρ is x=ρcosθ,y=ρsinθ.

The problem of noise removal can be transformed into a formulaic problem of solving the minimum value of Equation (15) on the image domain Ω. The equivalent Euler equation of ∂E/∂t=0 is taken as,

(16) $$\frac{\partial f}{\partial u}-\frac{\partial u}{\partial x}\left(\frac{\partial f}{\partial u_{x}}\right)-\frac{\partial u}{\partial y}\left(\frac{\partial f}{\partial u_{y}}\right)+\frac{\partial^{2}}{\partial x^{2}}\left(\frac{\partial f}{\partial u_{xx}}\right)+\frac{\partial^{2}}{\partial x\partial y}\left(\frac{\partial f}{\partial u_{xy}}\right)+\frac{\partial^{2}}{\partial y^{2}}\left(\frac{\partial f}{\partial u_{yy}}\right)=0$$

where,

(17) $$\begin{array}{ll} f & =\frac{1}{2}{\left|\frac{\partial^{2}u}{\partial \rho^{2}}\right|}^{2}=\frac{1}{2}{\left(u_{xx}\cos^{2}\theta +2u_{xy}\cos\theta \sin\theta +u_{yy}\sin^{2}\theta \right)}^{2}+\frac{\lambda }{2}{\left(u_{0}-u\right)}^{2}\\ \frac{\partial f}{\partial u} & =-\lambda \left(u_{0}-u\right),\;\frac{\partial f}{\partial u_{x}}=0,\;\frac{\partial f}{\partial u_{y}}=0,\\ \frac{\partial f}{\partial u_{xx}} & =u_{xx}\cos^{4}\theta +2u_{xy}\cos^{3}\theta \sin\theta +u_{yy}\cos^{2}\theta \sin^{2}\theta ,\\ \frac{\partial f}{\partial u_{yy}} & =u_{yy}\sin^{4}\theta +2u_{xy}\cos\theta \sin^{3}\theta +u_{xx}\cos^{2}\theta \sin^{2}\theta ,\\ \frac{\partial f}{\partial u_{xy}} & =2u_{xx}\cos^{3}\theta \sin\theta +2u_{yy}\cos\theta \sin^{3}\theta +4u_{xy}\cos^{2}\theta \sin^{2}\theta . \end{array}$$

Substituting Equation (17) into Equation (16), we can write,

(18) $$\begin{array}{l} \lambda \left(u_{0}-u\right)+u_{xxxx}\cos^{4}\theta +u_{yyyy}\sin^{4}\theta +u_{yyxx}\cos^{2}\theta \sin^{2}\theta +u_{xxyy}\cos^{2}\theta \sin^{2}\theta +2u_{xyxx}\cos^{3}\theta \sin\theta +\\ 2u_{xyyy}\cos\theta \sin^{3}\theta +2u_{xxxy}\cos^{3}\theta \sin\theta +2u_{yyxy}\cos\theta \sin^{3}\theta +4u_{xyxy}\cos^{2}\theta \sin^{2}\theta =0 \end{array}$$

The improved fourth-order OPDEs filtering model while using the gradient decline method is presented,

(19) $$\begin{array}{l} \frac{\partial f}{\partial u}=\lambda \left(u_{0}-u\right)+u_{xxxx}\cos^{4}\theta +u_{yyyy}\sin^{4}\theta +u_{yyxx}\cos^{2}\theta \sin^{2}\theta +u_{xxyy}\cos^{2}\theta \sin^{2}\theta +\\ \;2u_{xyxx}\cos^{3}\theta \sin\theta +2u_{xyyy}\cos\theta \sin^{3}\theta +2u_{xxxy}\cos^{3}\theta \sin\theta +2u_{yyxy}\cos\theta \sin^{3}\theta +\\ \;4u_{xyxy}\cos^{2}\theta \sin^{2}\theta \end{array}$$

The controlled diffusion factor $g\left(\left|\nabla u\right|\right)=1/\left(1+k{\left|\nabla u\right|}^{2}\right)$ is introduced to the improved fourth-order OPDEs model. The value of *g* becomes larger along the gradient direction, while it becomes smaller along other directions. Thus, the controlled diffusion factor can control the filtering speed. Equation (19) can be rewritten as,

(20) $$\frac{\partial f}{\partial u}=-g\left(\left|\nabla u\right|\right)\left(\begin{array}{l} \lambda \left(u_{0}-u\right)+u_{xxxx}\cos^{4}\theta +u_{yyyy}\sin^{4}\theta +u_{yyxx}\cos^{2}\theta \sin^{2}\theta +u_{xxyy}\cos^{2}\theta \sin^{2}\theta +\\ 2u_{xyxx}\cos^{3}\theta \sin\theta +2u_{xyyy}\cos\theta \sin^{3}\theta +2u_{xxxy}\cos^{3}\theta \sin\theta +2u_{yyxy}\cos\theta \sin^{3}\theta +\\ 4u_{xyxy}\cos^{2}\theta \sin^{2}\theta \end{array}\right)$$

Let (i,j) be any point on the image and the time step is Δt. Denote *p* by the number of iteration. In the evolution, evolutionary image $u\left(i,j,t_{p}\right)$ at $t_{p}=p\Delta t$ is presented as ${\left(u\right)}_{{}_{i,j}}^{{}^{p}}$, which can be expressed as

(21) $${\left(u\right)}_{{}_{i,j}}^{{}^{p}}=\frac{u_{{}_{i,j}}^{{}^{p+1}}-u_{{}_{i,j}}^{{}^{p}}}{\Delta t}$$

The discrete form of Equation (20) is

(22) $$u_{{}_{i,j}}^{{}^{p+1}}=u_{{}_{i,j}}^{p}-\Delta tg_{{}_{i,j}}^{p}\left[\begin{array}{l} {\left(u_{xxxx}\right)}_{{}_{i,j}}^{p}\cos^{4}\left(\theta_{i,j}\right)+{\left(u_{yyyy}\right)}_{{}_{i,j}}^{p}\sin^{4}\left(\theta_{i,j}\right)+{\left(u_{yyxx}\right)}_{{}_{i,j}}^{p}\cos^{2}\left(\theta_{i,j}\right)\sin^{2}\left(\theta_{i,j}\right)+\\ {\left(u_{xxyy}\right)}_{{}_{i,j}}^{p}\cos^{2}\theta \sin^{2}\theta +2{\left(u_{xyxx}\right)}_{{}_{i,j}}^{p}\cos^{3}\left(\theta_{i,j}\right)\sin\left(\theta_{i,j}\right)+\\ 2{\left(u_{xyyy}\right)}_{{}_{i,j}}^{p}\cos\left(\theta_{i,j}\right)\sin^{3}\left(\theta_{i,j}\right)+2{\left(u_{xxxy}\right)}_{{}_{i,j}}^{p}\cos^{3}\left(\theta_{i,j}\right)\sin\left(\theta_{i,j}\right)+\\ 2{\left(u_{yyxy}\right)}_{{}_{i,j}}^{p}\cos\left(\theta_{i,j}\right)\sin^{3}\left(\theta_{i,j}\right)+4{\left(u_{xyxy}\right)}_{{}_{i,j}}^{p}\cos^{2}\left(\theta_{i,j}\right)\sin^{2}\left(\theta_{i,j}\right) \end{array}\right]$$

where,

$${\left(u_{x}\right)}_{{}_{i,j}}^{{}^{p}}=\frac{u_{{}_{i+1,j}}^{{}^{p}}-u_{{}_{i-1,j}}^{{}^{p}}}{2},\;{\left(u_{y}\right)}_{{}_{i,j}}^{{}^{p}}=\frac{u_{{}_{i,j+1}}^{{}^{p}}-u_{{}_{i,j-1}}^{{}^{p}}}{2},\;{\left(u_{xy}\right)}_{{}_{i,j}}^{{}^{p}}=\frac{u_{{}_{i+1,j+1}}^{{}^{p}}-u_{{}_{i+1,j-1}}^{{}^{p}}-u_{{}_{i-1,j+1}}^{{}^{p}}-u_{{}_{i-1,j-1}}^{{}^{p}}}{4},$$

$$\begin{array}{l} {\left(u_{xx}\right)}_{{}_{i,j}}^{{}^{p}}=u_{{}_{i+1,j}}^{{}^{p}}-2u_{{}_{i,j}}^{{}^{p}}+u_{{}_{i-1,j}}^{{}^{p}},\;{\left(u_{yy}\right)}_{{}_{i,j}}^{{}^{p}}=u_{{}_{i,j+1}}^{{}^{p}}-2u_{{}_{i,j}}^{{}^{p}}+u_{{}_{i,j-1}}^{{}^{p}}\\ {\left(u_{xxxx}\right)}_{{}_{i,j}}^{{}^{p}}={\left(u_{xx}\right)}_{{}_{i+1,j}}^{{}^{p}}-2{\left(u_{xx}\right)}_{{}_{i,j}}^{{}^{p}}+{\left(u_{xx}\right)}_{{}_{i-1,j}}^{{}^{p}},\;{\left(u_{yyyy}\right)}_{{}_{i,j}}^{{}^{p}}={\left(u_{yy}\right)}_{{}_{i,j+1}}^{{}^{p}}-2{\left(u_{yy}\right)}_{{}_{i,j}}^{{}^{p}}+{\left(u_{yy}\right)}_{{}_{i,j-1}}^{{}^{p}}\\ {\left(u_{yyxx}\right)}_{{}_{i,j}}^{{}^{p}}={\left(u_{yy}\right)}_{{}_{i+1,j}}^{{}^{p}}-2{\left(u_{yy}\right)}_{{}_{i,j}}^{{}^{p}}+{\left(u_{yy}\right)}_{{}_{i-1,j}}^{{}^{p}},{\left(u_{xxyy}\right)}_{{}_{i,j}}^{{}^{p}}={\left(u_{xx}\right)}_{{}_{i,j+1}}^{{}^{p}}-2{\left(u_{xx}\right)}_{{}_{i,j}}^{{}^{p}}+{\left(u_{xx}\right)}_{{}_{i,j-1}}^{{}^{p}}\\ {\left(u_{xyxx}\right)}_{{}_{i,j}}^{{}^{p}}={\left(u_{xy}\right)}_{{}_{i+1,j}}^{{}^{p}}-2{\left(u_{xy}\right)}_{{}_{i,j}}^{{}^{p}}+{\left(u_{xy}\right)}_{{}_{i-1,j}}^{{}^{p}},{\left(u_{xyyy}\right)}_{{}_{i,j}}^{{}^{p}}={\left(u_{xx}\right)}_{{}_{i,j+1}}^{{}^{p}}-2{\left(u_{xy}\right)}_{{}_{i,j}}^{{}^{p}}+{\left(u_{xy}\right)}_{{}_{i,j-1}}^{{}^{p}} \end{array}$$

$$\begin{array}{l} {\left(u_{xxxy}\right)}_{{}_{i,j}}^{{}^{p}}=\frac{{\left(u_{xx}\right)}_{{}_{i+1,j+1}}^{{}^{p}}-{\left(u_{xx}\right)}_{{}_{i+1,j-1}}^{{}^{p}}-{\left(u_{xx}\right)}_{{}_{i-1,j+1}}^{{}^{p}}+{\left(u_{xx}\right)}_{{}_{i-1,j-1}}^{{}^{p}}}{4},\\ {\left(u_{yyxy}\right)}_{{}_{i,j}}^{{}^{p}}=\frac{{\left(u_{yy}\right)}_{{}_{i+1,j+1}}^{{}^{p}}-{\left(u_{yy}\right)}_{{}_{i+1,j-1}}^{{}^{p}}-{\left(u_{yy}\right)}_{{}_{i-1,j+1}}^{{}^{p}}+{\left(u_{yy}\right)}_{{}_{i-1,j-1}}^{{}^{p}}}{4},\\ {\left(u_{xyxy}\right)}_{{}_{i,j}}^{{}^{p}}=\frac{{\left(u_{xy}\right)}_{{}_{i+1,j+1}}^{{}^{p}}-{\left(u_{xy}\right)}_{{}_{i+1,j-1}}^{{}^{p}}-{\left(u_{xy}\right)}_{{}_{i-1,j+1}}^{{}^{p}}+{\left(u_{xy}\right)}_{{}_{i-1,j-1}}^{{}^{p}}}{4}. \end{array}$$

**Figure 4:**
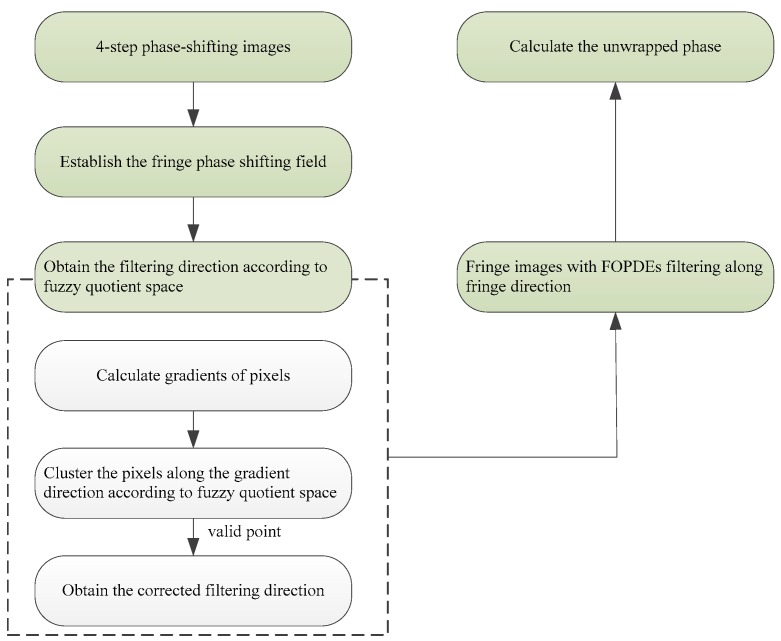
The flowchart of the fuzzy quotient space- oriented partial differential equations filtering (FOPDEs) method.

Figure 4 shows the flowchart of the proposed FOPDEs method.

The steps of the FOPDEs methods can be written as: (1) The four-step phase-shifting fringe patterns are applied to establish the fringe phase-shifting field. (2) For each phase-shifting image, the pixels along the fringe direction are classified into weak noised points and strong noised points by the fuzzy quotient space, and the modified filtering directions of strong noised points are calculated in the fringe phase-shifting field. (3) The improved fourth-order oriented partial differential equations with fidelity item is used to remove the Gaussian noise. (4) Phase extraction with filtered fringe images. In the next section, we will introduce the performance of the proposed method in detail.

## 4. Experiments and Results

In this section, we test our method on computer-simulated and experimental fringe patterns to verify the performance of our proposed method. All of the simulations listed here are implemented in Matlab R2018b on a laptop that was equipped with 3.0 GHz CPU and 8G RAM memory. 

**Figure 5:**
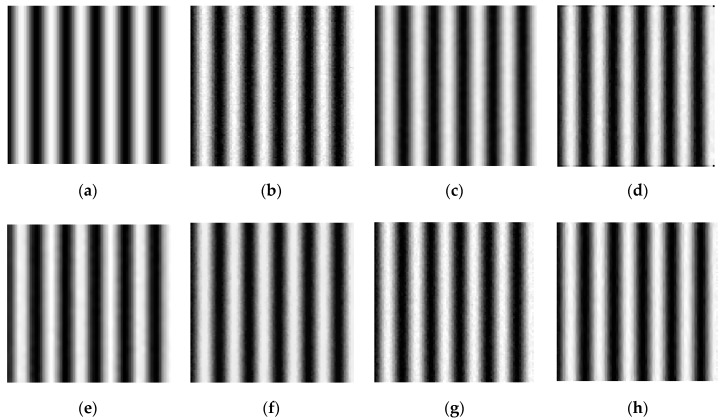
Fringe patterns with various filtering methods. (**a**) Noise-free fringe pattern, (**b**) noised fringe pattern with $\sigma^{2}=0.05\%$, (**c**) fringe pattern with Gaussian filtering, (**d**) fringe pattern with median filtering, (**e**) fringe pattern with wavelet transform, (**f**) fringe pattern with TV, (**g**) fringe pattern with OPDEs, and (**h**) fringe pattern with FOPDEs.

Traditional filtering methods only use PSNR as filtering evaluation index, which is difficult to fully reflect the comprehensive performance for phase-shifting images. Various noise variances are added to the four-step phase-shifting simulation images in order to verify the effectiveness of the proposed method. The PSNR and the standard deviation of phase error that are caused by Gaussian noise (STD) are used as indicators for filtering evaluation. The proposed FOPDEs method is compared with the commonly used methods, such as Gaussian filtering [16,17,18,21], mean filtering [16,17,18], wavelet transform [17], TV [19,20], and OPDEs [24], as shown in Table 1. Table 1 reflects the results of adding uniform noise to phase-shifting images and adding different noise to different phase-shifting images. Figure 5 shows the filtering effects of the 3th step fringe image (with size of 100 × 100) with various filtering methods.

As can be seen from Figure 5 and Table 1, with the increase of noise variance, the PSNR of Gaussian filtering, TV, OPDEs, and the proposed FOPDEs method tend to uniformly decrease, which shows that noise variance has an important impact on the filtering effect. Under different noise variances, the PSNR of median filtering does not change much, and it is significantly lower than that of the noisy image. This shows that the median filtering not only does not improve the filtering effect, but it also reduces the PSNR and increases the phase error. After Gaussian filtering, when the noise variance is strong (≥0.05%), the noise can be effectively filtered, but the edge blurred phenomenon appears. While, when the noise variance is weak (<0.05%), the PSNR will be reduced after Gaussian filtering. Gaussian filtering and median filtering belong to isotropic filtering. The fringe edges become blurred while filtering, which easily leads to a phase shift. Similarly, after wavelet transform, although the visual observation effect is better, with the increase of noise variance, the PSNRs, and STDs have little change and they are obviously lower than the noisy image. The TV method is superior to the wavelet transform method, but there has a ladder effect in the fringe pattern. The OPDEs method achieves a well filtering effect, and the PSNR is significantly improved. When compared with other methods, the proposed FOPDEs method has the best filtering effect, which achieves the optimal PSNR and minimum STD for the above noise variances. When compared with the noisy images, the proposed method can increase the PSNR by 27.24% and STD by 44.39%.

**Figure 6:**
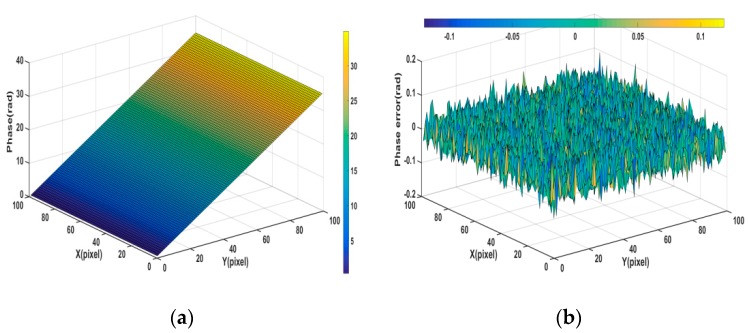

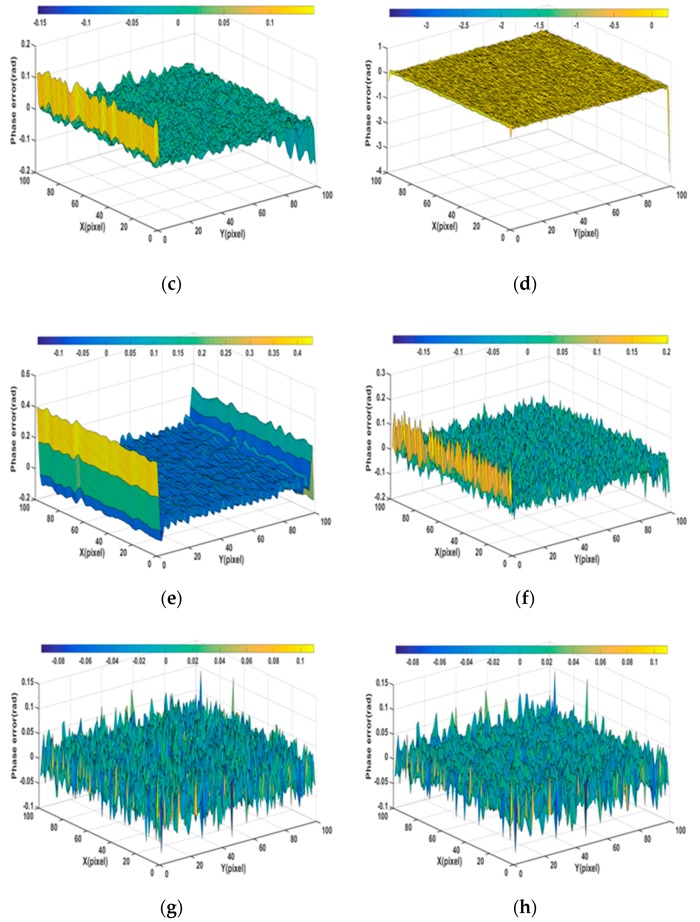
Phase error maps of simulated fringe image with various filtering methods. (**a**) Unwrapping phase, (**b**) phase error with noisy image, (**c**) phase error with Gaussian filtering, (**d**) phase error with median filtering, (**e**) phase error with wavelet, (**f**) phase error with TV, (**g**) phase error with OPDEs, and (**h**) phase error map with FOPDEs.

**Figure 7:**
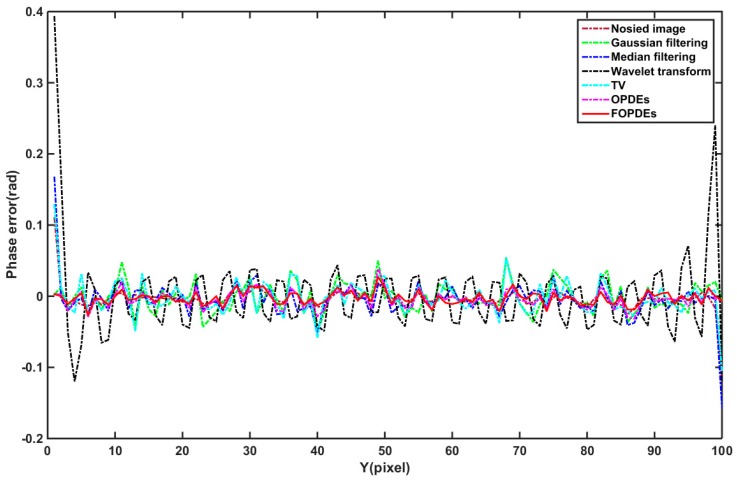
Phase error curves at the 20th row of Figure 6b–h with various filtering methods.

Figure 6 shows the phase error maps of the simulated fringe image that was filtered by various methods. From Table 1 and Figure 6b, we can see that, although the phase-shifting algorithm is noise resistant, the phase error that is caused by Gaussian noise still exists and it cannot be ignored. From Figure 6, we can see that Figure 6g,h are more close to the phase error distribution of the original noisy fringe images. According to the Table 1, as compared with OPDEs and other methods, FOPDEs has the smallest STD and highest PSNR. From Table 1 and Figure 6, the FOPDEs method retains more original image details than other methods. Figure 7 shows the phase error curves at the 20th row of Figure 6b–h with various filtering methods. As can be seen from Figure 7, when compared with other methods, the phase error curve with the proposed FOPDEs method is more close to 0, and it has the smallest error values. According to Figure 6 and Figure 7, there are edge effects in the Gaussian filtering, mean filtering, wavelet transform, and TV method. The phase error distribution with the Gaussian filtering, mean filtering, and wavelet transform vary greatly from that of original noised image. The TV method retains the phase error distribution characteristics of original noised image. From Figure 6 and Figure 7, although the curve fluctuation after Gaussian filtering is also smaller, the phase error with Gaussian filtering changes the characteristics of the original phase error map. The OPDEs method obviously retains more original image details than TV. As the fidelity item is added to the energy function of FOPDEs, the proposed method retains most of original image information. As can be seen from Table 1 and Figure 5, Figure 6 and Figure 7, the proposed method achieves optimal filtering performance while retaining more details.

**Figure 8:**
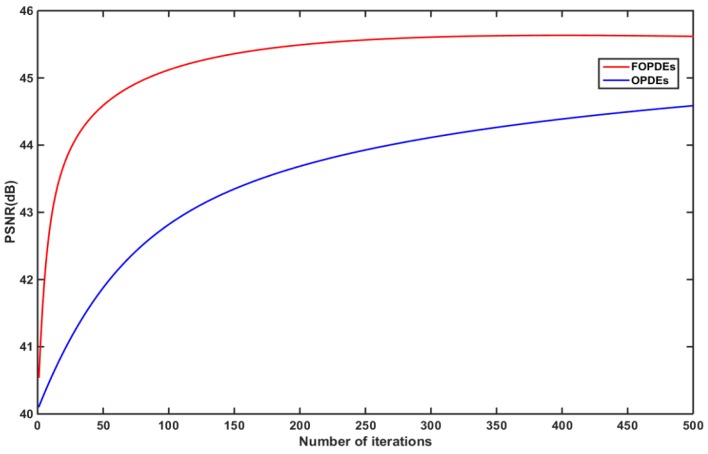
PSNR iteration-convergence curves with OPDEs method and FOPDEs method.

Figure 8 shows the convergence speed and algorithm performance of the proposed FOPDEs method. The FOPDEs method has reached the optimum PSNR value after 250 iterations, which means that the FOPDEs method is superior to OPDEs in convergence speed and in improving PSNR. Through the simulated fringe images experiment, we can see that the FOPDEs method improves the peak signal-to-noise ratio of the fringe images and reduces the standard deviation of phase error that is caused by Gaussian noise. At the same time, the FOPDEs method has faster convergence speed than the traditional OPDEs method.

**Figure 9:**
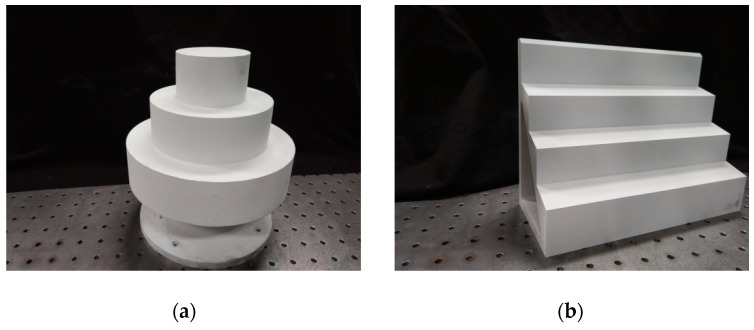
Stepped parts. (**a**) Multi-step cylinder part, and (**b**) multi-step plane part.

**Figure 10:**
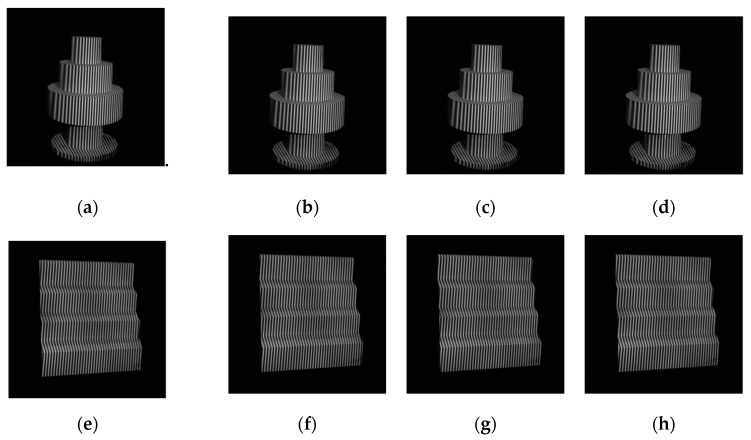
Fringe images. (**a**–**d**) are four-step fringe images of multi-step cylinder part, respectively, (**e**–**h**) are four-step fringe images of multi-step plane part, respectively.

The validity of the proposed method has been verified above by the simulated fringe images. The actual fringe images will be further validated. Figure 9 shows two actual stepped parts. Figure 9a is a coaxial cylinder part with several different diameters. Figure 9b is a part with multiple steps. The surfaces of both parts belong to a diffuse surface. Figure 10 shows the four-step phase-shifting images of the two stepped parts.

**Figure 11:**
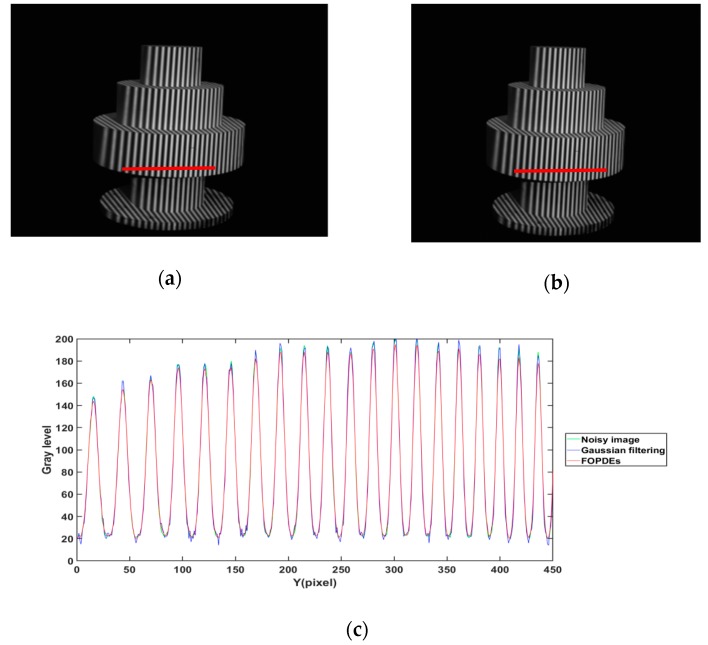
Fringe images of Figure 10c filtered by different filtering methods. (**a**) Fringe image with Gaussian filtering, (**b**) fringe image with FOPDEs, (**c**) cross sections of fringe intensity curves of (**a**,**b**) and the noisy image.

**Figure 12:**
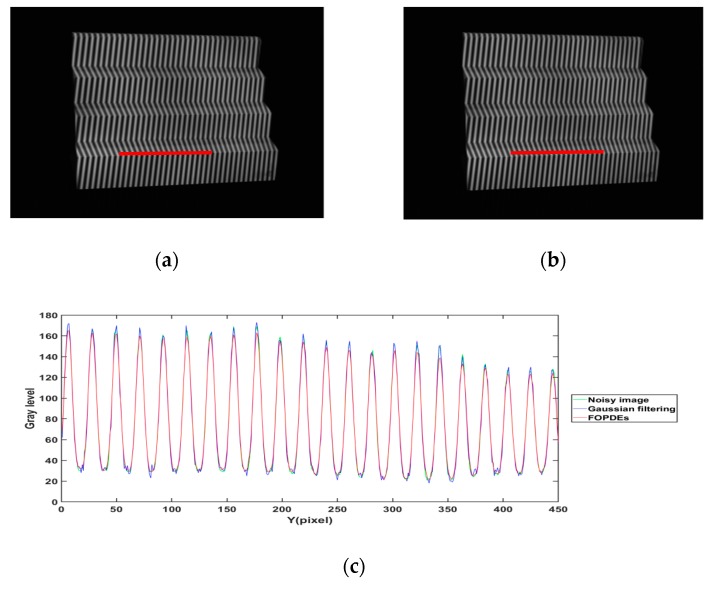
Fringe images of Figure 10g filtered by different filtering methods. (**a**) Fringe image with Gaussian filtering, (**b**) fringe image with FOPDEs, (**c**) cross sections of fringe intensity curves of (**a**,**b**) and the noisy image.

Figure 11 and Figure 12 show the filtering effect with Gaussian filtering and FOPDEs method for Figure 10c,g, respectively, in order to verify the filtering effect of the FOPDEs method. Figure 11c and Figure 12c show the cross sections of fringe intensity at the row 800th and the 500–950th columns of Figure 11a,b and Figure 12a,b, respectively. According to Figure 11c and Figure 12c, due to the effect of Gaussian noise, the cross sections of original fringe images show unideal sine curves. After Gaussian filtering, the valleys of the cross sections obviously fluctuate, which shows that the Gaussian filtering method has obvious deviation when it acts on the low gray values. The Gaussian filtering method is easy to cause phase shifting, which results in measurement error, while the FOPDEs method belongs to zero-phase-shifting filtering. When compared with the original fringe images and Gaussian filtering images, the FOPDEs method reduces the effect of Gaussian noise.

**Figure 13:**
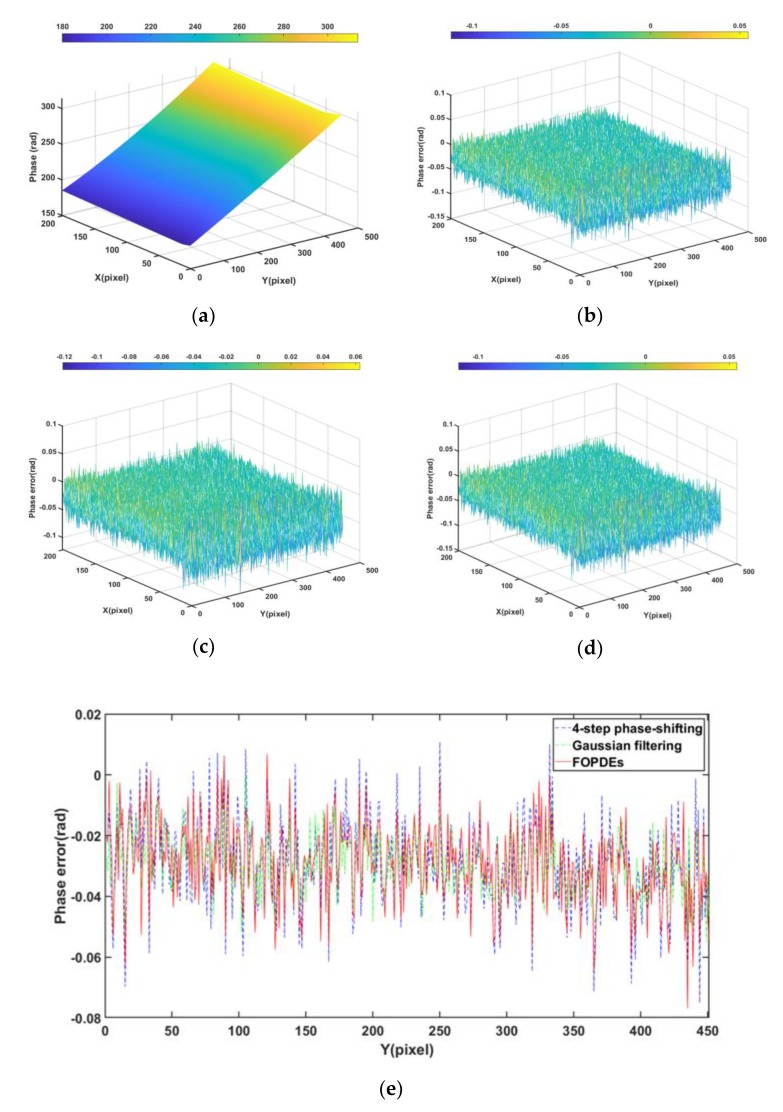
Phase maps of multi-step cylinder part. (**a**) Real phase unwrapping map with 16-step phase-shifting algorithm, (**b**) phase error obtained by four-step phase-shifting algorithm, (**c**) phase error obtained by four-step phase-shifting algorithm with Gaussian filtering, (**d**) phase error obtained by four-step phase-shifting algorithm with FOPDEs, and (**e**) phase error curves at the 100th row of (**c**,**d**).

**Figure 14:**
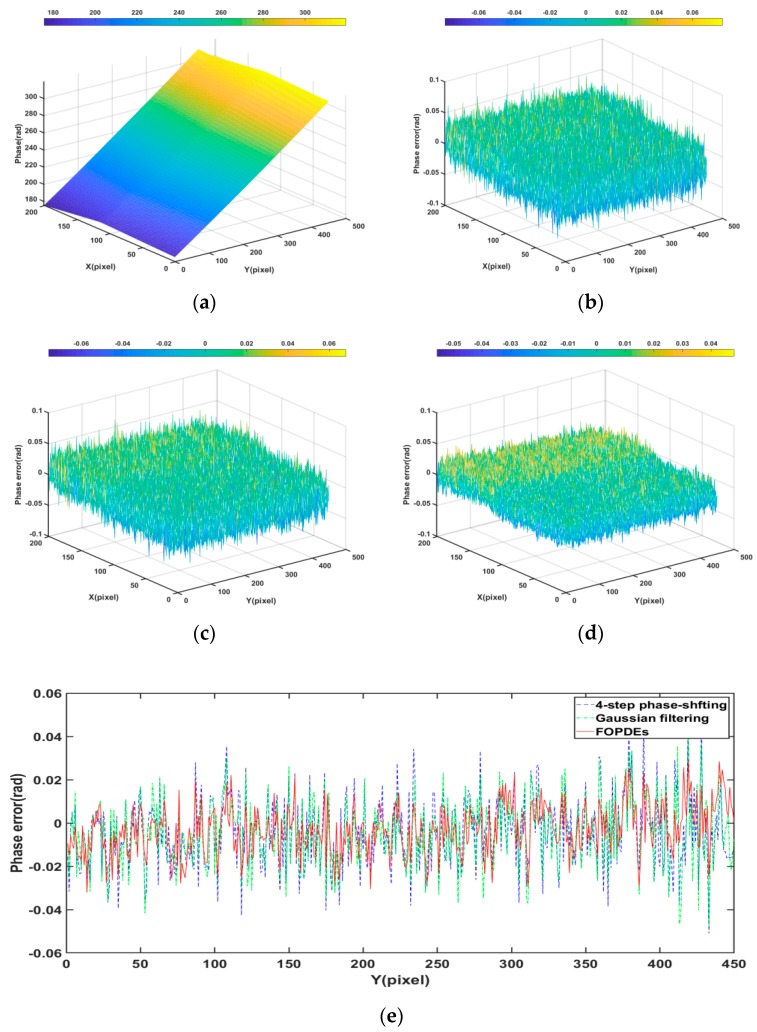
Phase maps of multi-step plane part. (**a**) Real phase unwrapping map with 16-step phase-shifting algorithm, (**b**) phase error obtained by four-step phase-shifting algorithm, (**c**) phase error obtained by four-step phase-shifting algorithm with Gaussian filtering, (**d**) phase error obtained by four-step phase-shifting algorithm with FOPDEs, and (**e**) phase error curves at the 100th row of (**c**,**d**).

The four-step phase shifting with non-filtering (four-step) method, four-step phase shifting with Gaussian filtering (Gaussian filtering), and four-step phase shifting with FOPDEs filtering method (FOPDEs) are used to process the fringe images above in Figure 10, respectively, in order to verify the filtering effect on phase error of the FOPDEs method proposed in this paper again, taking STD as the evaluation index. Subsequently, we calculate the STD results and the Mean STD values under different methods 10 times, respectively, as shown in Table 2. It can be seen that the FOPDEs method obtained the optimal results for both parts, which shows the excellence and stability. According to Equation (5), when the number *N* of phase shifting step is infinite, the phase error that is caused by noise can be neglected. Therefore, we choose the phase that was obtained by the 16-step phase-shifting algorithm as the real phase value of the object. Gaussian filtering and the proposed FOPDEs method process the four-step phase-shifted images respectively, in order to facilitate comparison. In order to better display the experimental results, we randomly choose the phase region for comparison, which is the 700–900th rows and the 500–950th columns of both phase error maps. When compared with the real phase, the phase error maps can be shown in Figure 13 and Figure 14 for both parts.

According to Figure 11, Figure 12, Figure 13 and Figure 14, the FOPDEs method can not only suppress the Gaussian noise of single fringe image, but also reduce the phase error that is caused by the Gaussian noise. From Figure 13 and Table 2, if the four-step phase-shifting algorithm was used, mean STD is 0.0165 rad, while Gaussian filtering, mean STD is 0.0151 rad. If the four-step phase-shifting images were filtered by FOPDEs, the mean STD is 0.0124 rad, which means that the FOPDEs method reduces the mean STD by 24.8% and 17.88% as compared with only the four-step phase-shifting algorithm and Gaussian filtering. From Figure 14 and Table 2, if the four-step phase-shifting algorithm was used, mean STD is 0.0178 rad, while the mean STD is 0.0163 rad after Gaussian filtering. If the four-step phase-shifting images were filtered by FOPDEs, mean STD is 0.0128 rad, which means that FOPDEs method reduces the mean STD by 28.1% and 21.47% as compared with only the four-step phase-shifting algorithm and Gaussian filtering. From Figure 11, Figure 12, Figure 13 and Figure 14, due to the phase error being not only affected by the noise, but also by the projection nonlinear effect and the influence of the phase unwrapping performance, the phase error is not completely eliminated, even if filtering. After Gaussian filtering, the distribution of the phase error is obviously different from the distribution of phase error with four-step phase-shifting, while the distribution of the phase error with FOPDEs is closer to the phase error distribution with four-step phase-shifting. The proposed FOPDEs method can effectively filter the Gaussian noise while keeping the detail information regarding the original fringe image better. Figure 13 and Figure 14 show the phase error curves with various methods. It can be seen that, after filtering with FOPDEs method, the phase errors have been reduced. It can be seen from Section 2 and Section 3 that, unlike the traditional single image filtering method, the proposed FOPDEs method belongs to multi-image filtering in fringe phase-shifting field, which requires complex operations, such as pixel direction, fuzzy hierarchical clustering, and fourth-order OPDEs filtering. The proposed FOPDEs method has the largest computational burden. The proposed method is more suitable for the situation of requiring higher measurement accuracy. Therefore, through the above simulated fringe images experiment and actual fringe images experiment, the proposed FOPDEs method can not only improve the PSNR and the standard deviation of phase error (STD), but also retain more details of original fringe images.

## 5. Conclusions

Fringe phase-shifting field based fuzzy quotient space-direction partial differential equations filtering method is proposed in order to reduce the phase error caused by Gaussian noise. Firstly, the concept of fringe phase-shifting field is established, transforming the independent filtering by traditional methods into multi-image filtering in phase-shifting field. Afterwards, the direction correction method that is based on fuzzy quotient space and the direction partial differential equations filtering method with fidelity item are proposed to adaptively determine the filtering direction and retain more details while smoothing the image. The proposed FOPDEs method can improve the PSNR through the experiments, and reduce the phase error caused by noise while retaining more details.

In future, the fringe image filtering method under various noises and how to extend the theory of fringe phase-shifting field to *N*-step (*N* > 4) phase shifting will be studied further. 

## Figures and Tables

**Table 1:** Comparisons of filtering results with different methods.

Noise Variance		0.0003%	0.01%	0.02%	0.03%	0.05%	0.1%	1*	2*
**Noisy Image**	PSNR	45.1694	39.9362	37.2023	35.3777	33.1817	30.3353	45.0698	40.0016
	STD	0.0081	0.0146	0.0207	0.0252	0.0327	0.0464	0.0080	0.0209
**Gaussian Filtering**	PSNR	34.3710	34.2658	34.2216	34.0142	33.6398	32.9800	34.3895	34.3190
	STD	0.0178	0.0182	0.0189	0.0194	0.0204	0.0222	0.0177	0.0185
**Median Filtering**	PSNR	32.1864	32.1748	31.9004	31.7873	31.3752	30.3924	32.1726	32.1758
	STD	0.0555	0.0562	0.0571	0.0542	0.0562	0.0558	0.0471	0.0530
**Wavelet Transform**	PSNR	31.0430	31.0081	30.9437	30.9519	30.8505	30.6780	31.0455	31.0030
	STD	0.0624	0.0625	0.0628	0.0628	0.0629	0.0633	0.0624	0.0626
**TV**	PSNR	34.0390	33.8797	33.6093	33.1969	32.5115	31.2737	34.1355	33.8892
	STD	0.0203	0.0218	0.0244	0.0262	0.0301	0.0375	0.0203	0.0242
**OPDEs**	PSNR	49.6068	44.6278	41.5815	39.9025	37.8154	34.8772	49.6381	44.6649
	STD	0.0069	0.0120	0.0169	0.0203	0.0264	0.0381	0.0069	0.0206
**FOPDEs**	PSNR	50.6144	45.7002	42.6183	40.8076	38.8956	38.5999	50.6905	45.4839
	STD	0.0048	0.0083	0.0115	0.0141	0.0185	0.0258	0.0047	0.0117

1*, 0.003%, 0.006%, 0.002%, 0.001% for four-step phase-shifting images, respectively. 2*, 0.01%, 0.003%, 0.05%, 0.02% for four-step phase-shifting images, respectively.

**Table 2:** Standard deviation of phase error (STD) results with different methods.

No.	Multi-Step Cylinder Part			Multi-Step Plane Part		
	Four-Step	Gaussian Filtering	FOPDEs	Four-Step	Gaussian Filtering	FOPDEs
1	0.0164	0.0150	0.0123	0.0177	0.0161	0.0125
2	0.0165	0.0155	0.0122	0.0174	0.0163	0.0127
3	0.0158	0.0153	0.0127	0.0174	0.0161	0.0127
4	0.0166	0.0149	0.0124	0.0179	0.0169	0.0125
5	0.0159	0.0148	0.0125	0.0181	0.0168	0.0131
6	0.0165	0.0157	0.0127	0.0179	0.0163	0.0129
7	0.0167	0.0155	0.0124	0.0183	0.0166	0.0130
8	0.0166	0.0148	0.0121	0.0185	0.0161	0.0128
9	0.0168	0.0149	0.0122	0.0179	0.0165	0.0129
10	0.0169	0.0148	0.0122	0.0171	0.0160	0.0127
Mean	0.0165	0.0151	0.0124	0.0178	0.0163	0.0128
